# Reflecting on the Plantationocene: the political economy of sugarcane plantations in Guangxi, China

**DOI:** 10.1080/03066150.2022.2087180

**Published:** 2022-06-27

**Authors:** Chunyu Wang, Yunan Xu

**Affiliations:** a College of Humanities and Development Studies (COHD), China Agricultural University, Beijing, People’s Republic of China; b Erasmus University Rotterdam International Institute of Social Studies, The Hague, Netherlands

**Keywords:** Plantationocene, sugarcane plantations, China, corporate control

## Abstract

This paper reflects on the concept of the Plantationocene through an analysis of sugarcane plantations in Guangxi province, China. It argues that although these plantations are owned and operated by local villagers, they are *de facto* controlled by corporations, and subject to state intervention through a ‘zoning scheme’. They are constructed and operated according to the same logic as other plantations all over the world, namely, *the logic of extraction based on cheap land and labor*. By demonstrating that plantations are not necessarily large-scale and do not always entail the alienation of land and labor, this paper hopes to empirically broaden the concept of the Plantationocene and to highlight the extractive nature of and the power relations around plantations.

## 1. Introduction

The term ‘Plantationocene’ was coined by Donna Haraway, Anna Tsing and other scholars during a conversation (Haraway 2015; Haraway et al. 2016; Mitman 2019), to refer to the ‘devastating transformation of diverse kinds of human-tended farms, pastures, and forests into extractive and enclosed plantations, relying on slave labor and other forms of exploited, alienated, and usually spatially transported labor’ (Haraway 2015, 162). The notion of the Plantationocene highlights the role of plantations in shaping and maintaining the hierarchical economic, social, political and cultural system. It centers on the logic of the plantation, including the processes of displacing the indigenous population, destroying original ecosystems and simplifying the landscape with monocrops, and involving highly racialized labor relations.

Recently, the concept of the Plantationocene has been further expanded and deepened by Wendy Wolford to understand plantations from three angles, namely, as a concrete and empirical social system; as a historically imperative form; and as a discursively ideal landscape (Wolford 2021). In this work, Wendy Wolford has engaged broad discussions around the political economic dynamics around and beyond plantations based on empirical studies conducted in the lusotropics (Brazil, Mozambique, and Portugal). In this paper, we focus on the empirical dimensions of plantations, particularly around the dynamic of the nexus of land and labor and the landscape that is shaped by contemporary plantations.

In the Plantationocene, plantations – as a prevalent form of production in the era of modernization – are normally cast in terms of large-scale monoculture under the firm control of corporations and involving the exploitation of labor (Wolford 2021). According to recent literature, such plantation-based production largely relies on the direct dispossession of local land users, as happened in the soybean and sugarcane plantations in Brazil (Borras et al. 2012; Rincón and Fernandes 2018; Ofstehage 2021) or the palm oil plantations in Indonesia (Li and Semedi 2021). To be specific, large areas of land are grabbed from villagers by corporations, combined and turned into plantations. At the same time, local villagers who were living off the land are either fully displaced or transformed into wage workers subject to unequal labor relations. Plantations thus always feature a simplified landscape and the alienation of land and labor.^1^

However, the sugarcane plantations in Guangxi province of China display distinctive dynamics in terms of landscape and land–labor relations. In Guangxi, sugarcane plantations are large in overall scale, but they are made up of many small-scale plots. The land for plantations is not seized by corporations but remains in the hands of local villagers and, instead of becoming plantation workers, local villagers are observed to farm these plantations, relying heavily on the employment of cross-boundary migrant labor. Why and how are these plantations constructed and operated in this way? Are these plantations inherently different from large-scale plantations elsewhere that involve the alienation of land and labor? And what are the implications of these plantations in terms of understanding the development of plantations worldwide?

To answer these questions, this paper explores the dynamics of the sugarcane plantations in Guangxi province. The analysis is mainly based on interviews with villagers, sugar company employees, migrant workers from Vietnam and state actors, which were conducted by the authors in F County and X County in Guangxi in the period 2014–2020.^2^ By directing attention to some distinct characteristics and trajectories of these plantations, the paper aims to call for a rethink of the nature of plantations. In this way, it directly engages into the discussions around the scale, dispossession, and extraction in the Plantationocene and thus hopes to contribute to a more nuanced understanding of the concept of the Plantationocene.

The remainder of the paper is organized as follows. The following section briefly introduces the general conditions of sugarcane plantations in Guangxi; this leads, in the third section, to a rethink of key features of plantations which involves a discussion around plantations worldwide. Following this discussion, section 4 analyses the key land and labor dynamics of the institutional settings in rural China that underpin the unique form of expansion of the Guangxi plantations. Section 5 takes a wider perspective on the features of plantations, and section 6 offers some concluding thoughts.

## 2. Sugarcane plantations in Guangxi

**Figure 1.**
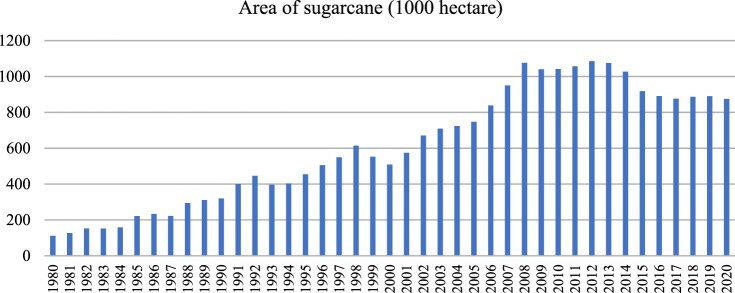
Area of sugarcane production in Guangxi (1980–2020). Source: National Bureau of Statistics.

While large-scale monoculture has also emerged and expanded in Guangxi, it has taken a different form than plantations in other regions around the world. As shown in Figure 1, the total area of sugarcane cultivation in Guangxi expanded approximately 10-fold from 111,000 ha in 1980–1.03 million ha in 2014.^3^ This scale of sugarcane plantations is comparable to, if not larger than, other regions.^4^

As in other parts of the world, sugar companies play a key role during the construction and operation of these sugarcane plantations. In Guangxi, there are 98 sugar companies, of which 28 are state-owned,16 are internationally owned and 54 are private (Wu 2020, 99). Among them, a Thai sugar company, East Asia Sugar Group, has received particular public attention due to the scale of capital involved and its transnational identity. The sugarcane produced in these plantations is mainly controlled by these companies for sugar production (field notes, 5 March 2015); the sugar produced in Guangxi accounts for more than 61.7% of the total sugar produced in China (National Bureau of Statistics). Sugarcane from Guangxi is also used, for example, in the production of fuel, fertilizer and monosodium glutamate (MSG), but these are byproducts of sugar processing and production quantities are currently low.^5^ Both sugar and other byproducts are mostly for domestic use rather than for export.

**Figure 2.**
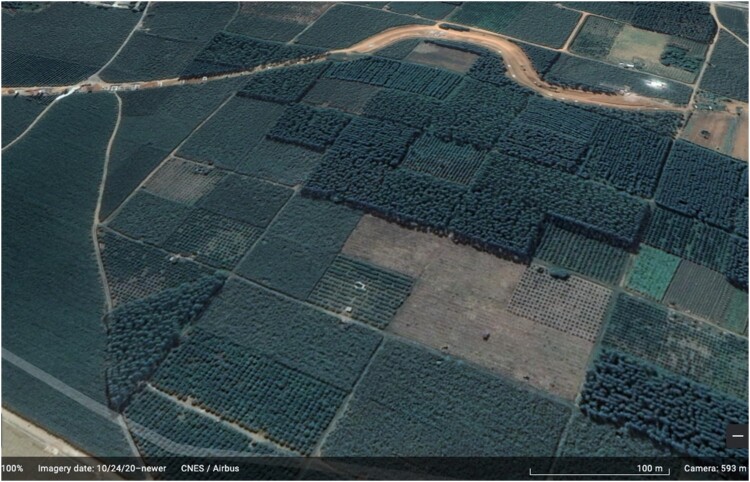
Patchwork landscape of sugarcane plantations in Guangxi. Source: google earth.

Taking a closer look at the plantations in Guangxi, however, several distinctive features become apparent. First, although the total area of the plantations is large, they are made up of segments that can be small – in many cases, as small as 1.3–2 ha.^6^ These plantations thus create a unique patchwork landscape as clearly shown in Figure 2. Moreover, the owners of these plantations, at least *de jure*, are still local villagers. In other words, local villagers have user rights over the farmland plots; they make the decision to plant sugarcane, although this land-use decision is strongly influenced by the state and corporate actors (sugar companies). The villagers themselves farm the sugarcane and own the outputs of the plantations, although the farming system relies on the large-scale supply of labor from Vietnam, and the planting, harvesting and marketing processes are *de facto* controlled by corporations – both state-owned and foreign sugar companies – under a special ‘zoning scheme’, which will be discussed further in section 4. This means that the villagers are neither deprived of the land they farm nor transformed into workers who are alienated from the outputs they produce, which is what has happened in most other plantations worldwide. This raises the question: can these sugarcane plantations that are small in scale, owned by local villagers and don’t involve land dispossession, still be considered ‘plantations’? To answer this question, the key features of a plantation should first be explored.

## 3. Rethinking plantations

Plantations are more than just a mode of production, but have loaded meanings. Wendy Wolford describes the plantation as ‘a set of social relations, an imperative, and an ideal that has endured around the world over the past 500 years’ (Wolford 2021, 1626). Similarly, Li and Semedi define the plantation as ‘a machine for assembling land, labor, and capital under centralized management for the purpose of making a profit; it is also a political technology that orders territories and populations, produces new subjects, and makes new worlds’ (Li and Semedi 2021, 1). In line with these definitions, plantations are understood as landscapes that are (re)shaping, and are (re)shaped by, land and labor relations under certain institutional settings, in a dynamic and relational way.

Plantations entail a large-scale simplification of the character of the landscape and involve massive displacements of populations and deployment of migrant labor. While this scenario captures the situation of many plantations around the world, it can only describe, rather than define, the current plantation system, because each plantation has its specific features (Li and Semedi 2021). This applies not only to the sugar plantations in Guangxi but also, for example, to the small-scale tree plantations in Vietnam which are operated by villagers and where there is no land dispossession (Sikor 2012). This implies that scale and dispossession may not be defining characteristics of plantations. Rather, when we trace their historical trajectory, we can say that plantations are extractive in nature and essentially entail investors’ firm control of large areas of *cheap land* and require stable supplies of *cheap labor*.^7^

Plantations are not a new form of production; their expansion is closely linked with colonialism. From the 17th to the 19th centuries, during the expansion of colonial power, vast swaths of land were acquired in the New World through enclosure and displacement, making way for the construction of plantations. Slaves and contracted coolie workers were sent to the plantations and became plantations workers under centralized control. Most of the outputs of plantations are exported to support Western industry directly or indirectly (Bhowmik 1980; Tsing 2012; Mintz 1986; Patel and Moore 2017). In this way, the violent land acquisitions were achieved at little and sometimes no economic cost, and the labor employed on the plantations was ‘forced through slavery, indenture’, and was thus rendered cheap, with a deliberately hierarchical system imposed (Tsing 2012, 148). In most plantations, workers are brought in from other regions, even when there is local labor available. This is not a coincidence but, as Haraway et al. (2016, 557) explicitly point out, is done ‘[b]ecause it is more efficient in the logic of the plantation system to exterminate the local labour and bring in labour from elsewhere. The plantation system depends on the relocation of the generative units: plants, animals, microbes, people. The systematic practice of relocation for extraction is necessary to the plantation system’. Tania Li also notes the preference of investors for long-distance recruitment, calling it ‘a reflection of the difficulty of extracting consistent, cheap labor from people who still have access to patches of land in the vicinity, hence other options’ (Li 2011, 286).

Nowadays, corporations – particularly international ones – have replaced the colonial powers to dominate plantations worldwide. These large-scale corporate-dominated plantations are ‘icons of modernity and orderly development, sometimes tinged with patriotic pride’ (Li and Semedi 2021, 2). In popular discourse, these plantations are framed in terms of ‘productive efficiency’ due to their concentrated and standardized production (Byerlee and Deininger 2013), although many scholars have argued that this might simply be the product of a speculative imagination (Tsing 2001) and that small farms tend to be more efficient on the ground, particularly in terms of the use of resources (Ploeg 2013). Meanwhile, in addition to big corporations, some individual capitalists, both external and local ones, are also observed to be important players in constructing and operating plantations. Compared with corporations, they are dominating plantations of a similar, if not larger, scale and their control power is not weaker and sometimes even stronger under certain contexts (Ofstehage 2018; Xu 2018).

Be that as it may, plantations have become a prevalent form of production following the recent waves of land grabs (Borras and Franco 2012; White et al. 2012). The establishment and operation of these plantations are often accompanied either by outright displacement or/and eviction of local communities, as with the oil palm plantations in Guatemala (Alonso-Fradejas 2012) or by the gradual exclusion and dispossession of smallholders, as with sugarcane plantations in Bolivia (McKay and Colque 2016). In some cases, local villagers are able to get employment on the plantations, although on adverse terms (McCarthy 2010), while in other cases, local villagers are totally excluded, as ‘their land is needed, but their labor is not’ (Li 2011, 286). These forms of exclusion represent the strategies of investors to reduce the costs of both land and labor through different institutional mechanisms. In other words, when the institutional setting means that it is cheaper to access land and labor by separating villagers from the land, corporations will tend to displace villagers and bring in labor from elsewhere. However, under other institutional conditions, this may be more difficult and even uneconomic; in those cases, investors may choose to control plantations indirectly (e.g. through contracts) and/or incorporate local villagers into their workforce, albeit on adverse terms.^8^

Investors’ firm control over large-scale cheap land and labor, in one form or another, is thus characteristic of contemporary plantations. Plantations in different regions tend to be constructed and operated in different ways to enable investors to extract with maximum effect under distinct institutional settings. In this sense, sugarcane plantations in Guangxi are *fundamentally* the same as other plantations all over the world. Although they are small scale per individual plot, and entail no direct dispossession by corporations, they are large scale in total, involve the mass employment of migrant workers from Vietnam, and thus become a vehicle for corporations to reap the lion’s share of profit with cheap land and labor. In addition, corporations are able to fully control the production, harvest and marketing processes of sugarcane under the zoning scheme. Thus, the distinctive dynamics of sugarcane plantations in Guangxi enable corporations to control large-scale land and sufficient labor for sugarcane production cheaply under the particular land and labor institutions in rural China, which will be elaborated in the following section.

## 4. Land and labor dynamics in the sugarcane plantations in rural Guangxi

In rural Guangxi, the unique dynamics of sugarcane plantations are (re)shaped by the institutional settings in rural China, including (1) the rural land system, which is closely associated with the patchwork landscape described above; (2) the zoning scheme, which enables tight corporate control over the production, harvest and marketing of the plantations; and (3) rural labor conditions, which lead to large-scale cross-border migration to fill the gaps in labor in the plantations, particularly during the cutting season. As a consequence of these circumstances, the transaction costs are too high for corporations to directly acquire and consolidate farmland for sugarcane production, which means that the sugar companies are better off leaving villagers on the land and incorporating them into the system. However, this does not mean that corporate control is less tight in these plantations. In order to fully control the production and the products of the plantations, instead of contract farming schemes which are common in other parts of the world, the zoning scheme is applied in Guangxi with the intervention of local state actors. On the other hand, there is a rural labor shortage in Guangxi because of the prevalence of internal migration. This makes it difficult for the sugar companies to extract stable and cheap labor from the villagers, who have more profitable livelihood opportunities. Thus, seasonal migrant workers from Vietnam, who come to the plantations either legally or illegally, have become a reliable source of cheap labor. These factors combine to explain the existence of sugarcane plantations that are individually small in scale, yet firmly controlled by sugar companies and employing substantial quantities of seasonal migrant labor from Vietnam. This section explores these issues in turn.

### 4.1. The landscape and rural land system

**Figure 3.**
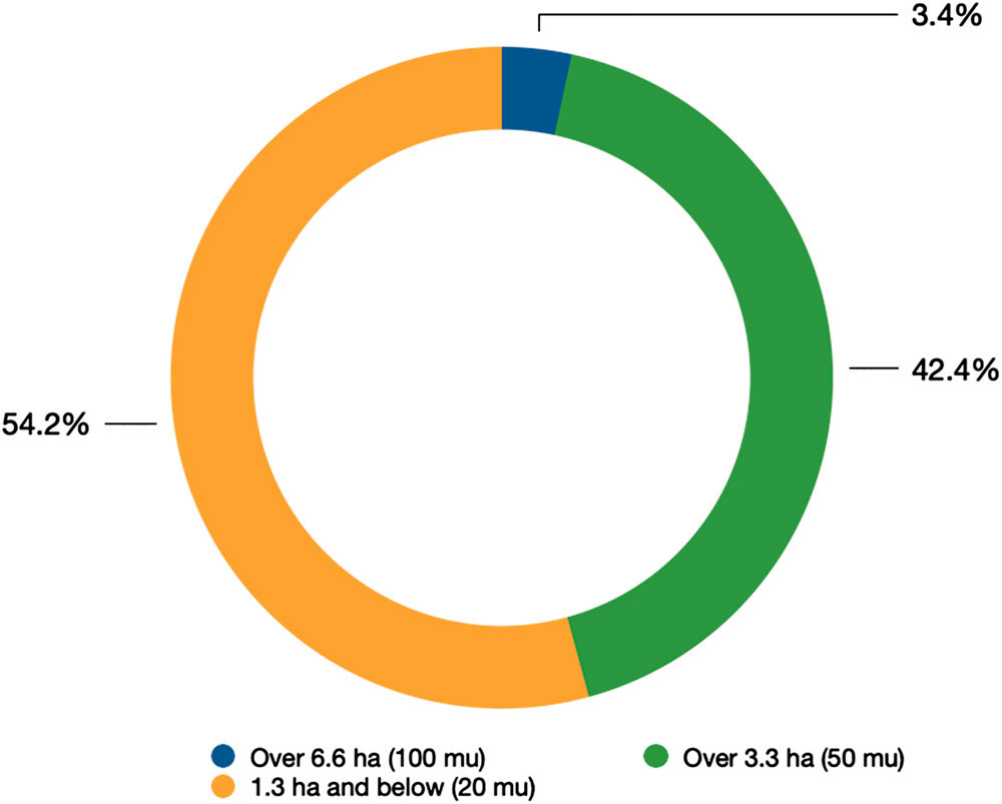
Distribution of farmland area in B Village. Notes: total farmland area of the village = 206.6 ha (3100 mu); total number of households = 118. Data source: Field notes, 8 January 2016.

In rural Guangxi, like other regions of China, farmland has been distributed to rural households under the Household Responsibility System (HRS) reform. Under the HRS reform, the user/management rights to farmland were separated from the property rights and distributed to rural households, while the property rights still belonged to the rural collectives (Ho and Spoor 2006; Ye 2015).^9^ Under the principle of fairness, farmland was usually first classified according to quality, and then divided and distributed based on the size of households (Unger 2002; Ye 2015). Thus, each rural household will be allocated several land plots, including both good and poor-quality land. However, households’ actual land access is highly differentiated because of varying family sizes and differing capabilities to reclaim land^10^ (as shown in Figure 3). In Guangxi province, a household might access as many as 24 land plots (field notes, 8 January 2016), leading to a fragmented landscape in rural Guangxi.

The fragmented landscape created by the land reform makes it difficult for investors to acquire and consolidate land to construct plantations, as they need to negotiate with many smallholders instead of a single holder. This increases the transaction costs for large-scale land acquisitions (Borras et al. 2018). In this sense, although the land property rights situation is not straightforward in rural China, given that the collective is quite an ambiguous unit (Ho 2001), villagers are not particularly vulnerable; rather, they have high levels of autonomy and capacity to retain their access to land under this land system.^11^ This resonates with Zhang and Donaldson’s (2008) argument that China’s rural land system actually hinders large-scale land dispossession. An example is provided by the experience of a domestic specialized enterprise which undertook an arduous land-leasing process in order to construct a large-scale sugarcane plantation in C City, under the ‘double high’ (*shuanggao*) program.^12^ When the enterprise came to lease farmland in one village in 2015, it needed to negotiate with and get approval from all 240 households in the village. In 2017, there were 20 households that had not yet signed the contract – and their plots were in the middle of the land that the enterprise wanted to rent. Thus, to consolidate the land in order to apply industrialized production methods, the company had no alternative but to persuade those villagers who did not want to lease land, to exchange their plots for plots on the edge of the area in question (field notes, 18 March 2017).

In addition to the complicated land-leasing process, rents for rural farmland are high. The villagers are in a relatively good negotiating position due to their clear land access rights under the land system, as confidently explained by a villager who has leased his farmland to a domestic specialized company for sugarcane production in X County:

The land rent is 1000 yuan per mu per month. It [the rent] is not fixed but will increase with the commodity price [here referring to the sugarcane price]. In the contract, the price of sugarcane was 450 yuan per ton. If the sugarcane price increases, the land rent will increase accordingly. If the price of sugarcane increases to 550 yuan per ton, then the land rent will change to 1100 yuan per mu per month. If the price of sugarcane decreases, the land rent will remain the same as 1000 yuan per mu per month. (Field notes, 15 March 2016)

Linking this land rent with the other costs entailed in large-scale sugarcane production (as shown in Table 1), it is estimated that the total cost of labor and other inputs for producing a truckload of sugarcane was 3915 yuan for 3 mu during the 2015/2016 cutting season in Guangxi (authors’ fieldwork in Guangxi, January 2016). Thus, the cost for producing sugarcane per mu (excluding land rent) was around 1305 yuan (3 divides 3915). Meanwhile, the purchase price of sugarcane was 440 yuan per ton at that time and the average output in Guangxi was 5 tons per mu. Deducting the costs for labor and other inputs, the profit for sugarcane production would be 895 yuan per mu. If the land rent is 1000 yuan per mu, then the net profit is −5 yuan per mu. This implies that gaining direct access to land to engage in sugarcane production would be uneconomic for corporations. This is reflected in the high levels of bankruptcy among enterprises that tried to consolidate land to operate large-scale sugarcane plantations in Guangxi, even when they were offered subsidies from the state under the ‘double high’ program (field notes, 10 June 2016).

**Table 1.** Labor and input costs for sugarcane production.

Cost	Task/Item	Amount	Unit	Total cost per truckload (equivalent to 3 mu)
Labor cost	Cutting	1	Yuan per bundle	1000
	Carrying	0.3	Yuan per bundle	300
	Loading	350	Yuan per truck	350
	Planting	110	Yuan per mu	330
	Fertilizing	1bag *10 yuan * 3 = 30	Yuan per mu	90
	*Subtotal*			2070
Cost of inputs	Rental of farm machinery	110	Yuan per mu	330
	Fertilizer	3*150 = 450	Yuan per mu	1350
	Herbicide	10	Yuan per mu	30
	Pesticide	45	Yuan per mu	135
	*Subtotal*			1845
**Total**				3915

Source: Fieldwork, January 2016.

Notes: This cost excludes land rents. There are 1000 bundles of sugarcane in a truck, equivalent to 15 tons, and produced from 3mu farmland. Price is for the 2015/2016 cutting season in F County. The labor cost was calculated based on the cheapest labor price when employing migrant labor from Vietnam.

There are also other costs involved in large-scale plantations, given the complicated social relations around land. For example, it was observed that villagers who worked in large-scale plantations directly controlled by a sugar company dug holes in the plantations and buried fertilizer there, which they later took home (field notes, 20 March 2017). Such pilfering is essentially one of the ‘weapons of the weak’ and part and parcel of conflicts between the company and villagers (Scott 1985). This hints at the high management costs for investors who attempt to directly control large-scale plantations.

Taking account of the above, it is more profitable for sugar companies in Guangxi to leave the villagers on the land and engage them in the production process. As an employee of a sugar company in Guangxi pointed out, ‘around 90% of the plantations in our zone [that supply raw materials for the sugar company] are individually owned and there are no [plantations owned by] specialized enterprises’ (field notes, 4 January 2016). Thus, eschewing further land consolidation, a unique patchwork landscape of sugarcane plantations emerges, which are individually small in scale, but add up to be large scale in total.

### 4.2. Land control and the zoning scheme

As the analysis above suggests, the rural land system seems be a barrier to the development of large-scale monoculture, given that direct land access appears to be uneconomic. The risks of contract farming are also high for corporations in rural China, given serious side-selling problems (Zhang 2012). This implies that a more innovative way to control the land use, production and outputs is required for sugar companies, both state-owned and transnational. In the context of Guangxi, this control is realized through the zoning scheme.

The zoning scheme was initiated by the local government at the city level in Guangxi in the early 1980s. This scheme was primarily set to strictly plan and control sugarcane production under the planned the economic system. But it remained in Guangxi even after the decline of planned economic systema regulation issued in 2002 (*the Interim Measures for Sugarcane Management*,糖料蔗管理暂行办法). According to our fieldwork in Guangxi, the measurements within this zoning scheme are generally unchanged, although the zone areas within each county might have been slightly adjusted due to the entry of new sugar companies.

Under this scheme, land within a county is divided into several zones. Inside a zone, all the sugarcane produced must be sold to a nearby sugar company, whether state-owned, private, or international (an example of zone areas in a country is shown in Table 2). This means that under this scheme producers are bound to a certain sugar company. On the one hand, sugar companies are obligated to offer support to producers in order to ensure a sufficient harvest, and to purchase whatever amount is produced in the zones that are allocated to them. On the other hand, the small-scale sugarcane plantations are firmly controlled by sugar companies under the zoning scheme. In other words, sugar companies have *de facto* control over the production, harvest and distribution of sugarcane in these plantations, without having direct ownership, via a set of measures as shown in Table 3.

**Table 2.** Zone areas for each sugar company in F County, 2020.

Zones	Areas (1000 mu)	Number of towns/townships covered
Zone 1 for a foreign company	570	8
Zone 2 for a private company	480	4
Zone 3 for a state-owned company	52	2
Zone 4 for a state farm	16.3	1

Source: Wu (2020, 92).

**Table 3.** Measures taken by sugar companies under the zoning scheme in Guangxi.

Process	Measures	Aims of corporations
**Production**	Subsidies for changing land use to sugarcane production Technical support (e.g., training, subsidies for plastic cover and related machines) ‘Lending’ seedlings and fertilizers Categorized price system	To ensure sufficient and high-quality supply of raw materials To standardize the production process
**Harvest**	Sugarcane tickets	To control the cutting process and ensure the steady, sustained, and timely supply of raw materials
**Distribution**	Setting price	To control outputs

Note: in Guangxi, one bundle = 15 kilogram.

Data source: field notes in 2016 and online interviews in 2020.

First, to control the production process, sugar companies offer subsidies for land-use change to sugarcane production; they provide technical support, ‘lend’ seedlings and fertilizers (which are paid for later, when the sugar is sold to the companies) and deploy a categorized pricing system for sugarcane of different qualities. To be specific, when villagers change their land use from the cultivation of banana or eucalyptus to sugarcane, sugar companies offer a subsidy of 400 yuan per mu. If the land use is changed from other crops to sugarcane production, the subsidy is 200 yuan per mu (field notes, 16 March 2016). In this way, sugar companies aim to compete with those who invest in other crops, particularly crops that have booming markets, in their effort to dominate the use of the land and to ensure a sufficient supply of raw materials. The sugar companies also offer training in sugarcane production and subsidies for villagers to acquire or rent plastic cover and related machines. While these measures seem to support the small-scale producers, they are primarily intended to achieve more control over the production process. As an employee at one of the sugar companies explained, ‘when using the cover [in the sugarcane plantation], the yield can increase by 1 ton per mu’ (field notes, 4 January 2016). Similarly, another employee told us:

Our [land] is distributed to each household. Land is managed by those sugarcane producers themselves. As long as the crop that a villager produces is legal, he/she can plant whatever he/she wants to plant. We sugar companies cannot force [them]. What we can do is to provide subsidies for them and offer good service to them, in order to encourage them to plant sugarcane … . The sugar company just wishes to use all the usable land to plant sugarcane. (Field notes, 4 January 2016)

Moreover, sugar companies supply seedlings and fertilizers to villagers to ensure the sugarcane produced in their zone will have the right inputs. In return, the small-scale planters are contracted to sell their produce to the sugar company that has supplied them, and the costs of seedlings and fertilizers are then directly deducted from the payment they receive. This is not a self-regulating process, but is closely monitored by local coordinators, who are normally village elites (e.g. village cadres) and employed by the sugar companies. If a villager who took seedlings and fertilizers from a sugar company does not deliver his/her outputs back to that company, the income of the coordinator in that village would be reduced. These dynamics thus confirm that the support offered by sugar companies during the production process is in fact in exchange for control over output.

**Figure 4.**
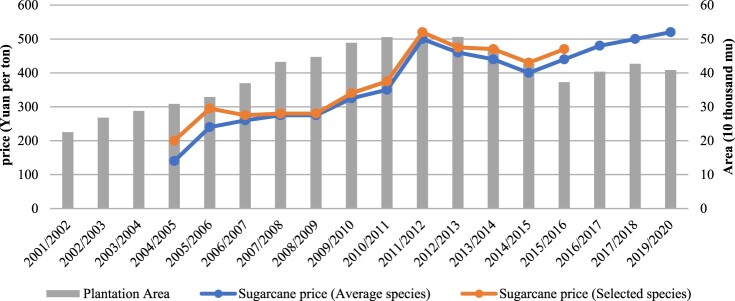
Sugarcane price and plantation area in Nanhua Mill, Guangxi (2001–2017). Data source: NanHua Sugar Mill (field notes in 2016, 2018 and online interviews in 2020).

Sugar companies also apply a graded pricing system when they purchase sugarcane from small-scale plantation owners. Sugarcane is categorized every year according to species, and the companies check and identify the sugarcane that is transported to processing mills. As shown in Figure 4, companies pay a higher price for sugarcane that is cultivated from selected species. These selected species have higher sugar content; they are not local, but are usually introduced by sugar companies from other regions (e.g. *Tai No 22* introduced from Taiwan). Under this pricing system, instead of saving seedlings in the traditional way, the small-scale producers have to purchase seedlings of recognized species, either from sugar companies or other market channels. The pricing system thus promotes the commodification of seeds, on the one hand, while it standardizes the production process in the plantations on the other hand. As a villager who planted 12 mu sugarcane told us, ‘[I] used to plant yellow sugarcane [a local species]. The sugar company said that it does not fit the standard. So [I] do not plant [this species] any more’ (interview, 10 June 2016). Similarly, another villager complained about the control exercised by the sugar company via this pricing system: ‘the species that were [recognized] as the first category last year might become the second category in the next year’ (interview, 10 June 2016). This clearly suggests that, rather than a concern for the welfare of the small-scale producers, a standardized and well-controlled production process is the real aim of the sugar companies. In other words, these measures are intended to ensure a sufficient and high-quality supply of raw materials for the companies’ production.

Second, to control the harvesting process, sugarcane ‘tickets’ (*zhepiao*) are deployed. Sugarcane is a crop that needs to be processed within a short time after cutting because the sugar content decreases rapidly. Each sugar company has capacity in its mills to process a certain amount of sugarcane daily after harvest. This means that if too much sugarcane is cut and exceeds the capacity of mills, sugarcane would be wasted; if not enough is cut, then part of the sugar companies’ costs for processing (labor cost, energy cost, etc.) would be wasted. Sugar companies therefore tend to have sugarcane in their zones cut and delivered according to a schedule. The company issues sugarcane tickets to plantation owners, which specify the amount of sugarcane and the date it is to be delivered to the mills. Only with a ticket, can the planters cut their sugarcane and transport it to the sugar mills. Without a ticket, their sugarcane would not be purchased by the sugar companies, and might be left to rot near the roadside. This situation is vividly explained by a villager: ‘without sugarcane ticket, [we] cannot cut sugarcane; [and] without sugarcane tickets means no money from sugarcane’ (field notes, 6 August 2016). In this way, sugar companies can determine whose sugarcane can be cut first and whose cannot, and can thus also determine who can gets paid first, and who does not. Thus, these tickets are not only a vehicle to control the small-scale sugarcane plantation owners in the zone, but they also become a source of competition among these owners, all hoping to have their sugarcane cut in time. In this way, sugar companies are able to control the cutting process across these scattered plantations and to ensure a steady, sustained and timely supply of raw materials to their mills.

Third, to ensure that all the sugarcane produced within the zone is sent to a certain sugar mill, a unified purchase price is set across the province. The lowest purchase price for sugarcane is determined by the local government at the provincial level each year, at the beginning of the crushing season, but it is coupled with the fluctuating price of sugar: when the price of sugar rises, the price of sugarcane increases accordingly.^13^ However, since the lowest price for the year has already been set, it will not change if the sugar price drops. The purchase price is standardized across the province, leading an official in Guangxi to remark that ‘sugar companies are lying on their zones and there is little competition among the sugar companies’ (field notes, 20 March 2017). This implies that the sugarcane market is not a liberalized market, and its price is relatively stable compared with other boom crops in the region (such as eucalyptus). Thus, although there are still variations in the price of sugarcane, as shown in Figure 4, many villagers continue to cultivate sugarcane on at least part of their land because, as a villager explained, ‘planting sugarcane is a hard work, but [the sector] is relatively stable and the income [from this sector] is secured’ (field notes, 19 March 2017). At the same time, the standardized purchase price across the region means that plantation owners are not eager to sell their outputs to other sugar companies. Nevertheless, we did hear of some cases of sugar companies, eager to increase production when the sugar price was rising, trying to lure sugarcane farmers outside their own cane zone to sell sugarcane to them, through a ‘cash for cane’ procurement policy. Farmers would get their money one or two days after the sugarcane was loaded onto the truck, instead of waiting for one month – or even several months – if they sold to the originally designated sugar company. This practice caused chaos and conflicts; moreover, since it meant that taxes would go with the sugarcane trucked out of the jurisdiction, the local government took actions to prevent it, sending out police and patrol cars to stop these sales (field notes, 6 March 2015).

In addition to the measures discussed above, sugar companies also attempted to directly control land within the zone under the ‘double high’ program to ensure the scale of operation. For example, a sugar company in Chongzuo city leased 6000 mu land from villagers. Instead of directly operating those plantations, the company released the land plots to four specialized companies and two individuals (*dahu*), to avoid the high costs and risks involved in sugarcane cultivation. According to an employee of the sugar company, the rent they charged the investors was the same as the rent they paid to the villagers (field notes 4 January 2016). This shows an explicit attempt of sugar companies to maintain control of sugarcane plantations in their zone. However, the endeavor failed: many investors walked away and abandoned the plantations. At the time of the research, one of the plantations had already been through the hands of three different investors (field notes, 30 January 2018).

Within this zoning scheme, the role of the state is critical. It is the local government that divides the land and distributes the zones to sugar companies. Moreover, the local state also sets the basic sugarcane purchase price every year, offers subsidies and tax benefits to sugar companies for introducing high-quality seedlings, and facilitates sugar companies in blocking the cross-zone sale of sugarcane (referred to as ‘black sugarcane’ by the employees of sugar companies). These state actions underpin the state’s deliberate efforts to control sugarcane as a strategic crop and a component of national sugar/food security.^14^ These measures stabilize and normalize the zoning regime and ensure corporate control over the sugarcane plantations.

In short, the zoning scheme allows sugar companies to maintain firm control over the scattered small-scale sugarcane plantations, via both economic and extra-economic means and facilitated by the local state. Those measurements taken by sugar companies are essentially based on those companies’ privileged access to financial capital and technology. Without needing to possess the land or operate the plantations, corporations have access to a stable, reliable, standardized and timely supply of the raw material they need. This might be considered a new form of coercion, albeit coercion without force, given that the lion’s share of the benefits accrues to the corporations.

### 4.3. Labor dynamics

In rural China, following the processes of urbanization and industrialization that began in the 1980s, villagers – especially the strong and young – have left the land on a massive scale, to seek off-farm work. Most migrate from rural to urban areas (leaving home) while a small proportion work locally (without leaving home) (Huang 2009; Ye et al. 2013). The great majority of those internal migrant workers (*nongmingong*) find work in the construction and manufacturing sectors. Compared with the income from farming, income from migrant work is much higher, as explained by a villager in Guangxi: ‘one day’s wage [from migrant work in urban areas] is already able to purchase two bags of rice’ (field notes, 23 February 2016). Jun Han, the deputy director of the Office of Rural Work Leading Group stated that: ‘Nowadays most of rural households have around 7–8 mu or 10 mu land … annual income from farming on such tiny land is almost the same as the income gained from wage work in urban areas in one week’ (Guo and Tong 2015).

Guangxi follows the same trends in labor dynamics. According to a survey conducted by the authors in Guangxi in 2016, around 90% of the 104 rural households surveyed were undertaking some non-agricultural work and about 80% of the households had at least one member migrating to urban areas to do wage work. In the rural households surveyed, 42.6% of the villagers aged 18–65 who were able to work were doing migrant work.

When most of the young and strong population are working in urban areas, only the very young, the weak and the old are left behind. This is linked with a trend of the ageing of local farming labor force (Rigg et al. 2020) and has caused a serious labor shortage in rural Guangxi. When the labor shortage is combined with the prevailing local cultivation of a labor-intensive crop, namely sugarcane, the situation is even worse. Sugarcane requires abundant labor, particularly during the cutting season. Once a plantation owner receives a sugarcane ticket from the sugar company, he/she needs to cut the sugarcane and deliver it to the mill for crushing within a very short period (usually one or two days). The huge demand for labor during the harvest often cannot be met through family labor alone, although many plantation owners try to manage the production process (including planting, weeding and fertilizing) with family labor. According to interviews in Guangxi, villagers who own 10 mu or more of sugarcane plantations need extra, external labor during the cutting season.

Before internal migration became so prevalent, labor exchange was used to fill the labor gap, but this can no longer provide a solution. A villager told us: ‘[we] will not exchange labour … even brother cannot help. When [we] are busy, we just hire labor’ (field notes, 10 June 2016). In the 1990s, these plantation owners started to employ outside labor. At first, they employed workers from the nearby counties, then workers from other provinces (e.g. Guizhou and Yunnan province).

More recently (before the Covid-19 pandemic), most of the workers employed in these plantations came from Vietnam, mainly from those villages near the China-Vietnam border (field notes, 9 March 2015). These Vietnamese workers’ migration to China is based on certain political economic conditions. On the one hand, these migrant workers could get around 3000 yuan (445 USD) per month on average from sugarcane cutting during the harvest season as of 2018 (field notes, 1 January 2018), while the average monthly salary/wage income in Vietnam was 5.36 million Vietnamese dong (231USD) in January 2018.^15^ This means that they can earn around twice in rural Guangxi as much as they could get inside Vietnam. Such economic gains motivate the flow of labor across the border and the income from seasonal work in the sugarcane plantations in Guangxi can offer a vital supplement to these migrant worker’ economic production and social reproduction at home in Vietnam, as what has happened to migrant workers from Myanmar (Borras et al. 2022). On the other hand, similar to the situation of China-Myanmar border, those migrant workers are able to enter China via both formal channels (e.g. marriage and applying for work permit in China) and informal channels (Borras et al. 2022; Hua et al. 2019; Baird and Cansong 2017).^16^ Such flow of labor across the border does play a key role in the recent development of sugarcane plantations in Guangxi.

The shift from exploiting local labor to employing external labor in those sugarcane plantations in Guangxi is closely linked with labor cost, as clearly shown in the following example of a rural household in D Township that owns 40 mu sugarcane plantations.

[We] hired workers after 1998. Workers were normally from A County, T County, B County and M County [nearby counties]. We all employ seasonal workers. No-one in the village employs long-term workers [in the sugarcane plantations] because the management of plantations like weeding can be conducted by a couple in the family … At that time [around 1998], [we] hired seven people, and they could cut one truck of sugarcane in one day. But in 2010, the wage [of the workers] from T County had reached 130/140 yuan per ton. Around 2011, [villagers] in the village started to hire Vietnam[ese] migrant workers … when hiring a Vietnam[ese] migrant worker, 50 yuan needs to be paid to a *Jiubalao* [a broker]. Now I employ nine Vietnamese migrant workers. They can cut a truck of sugarcane a day. Now the [cost of] labor hiring is all calculated based on the bundles [cut]. Vietnam[ese] migrant workers’ bundle is 30 jin per bundle while workers from T[County] is 23–24 jin per bundle. [The costs for the two groups of workers] are both 1 yuan per bundle. Thus, hiring T [County] workers is more expensive. Now [villagers in the village] all employ Vietnam[ese] migrant workers. No one employs workers from T[County] … there are more than 20 groups of Vietnam[ese] workers in the village. Each group has seven/eight people. (Field notes, 5 January 2016)

This change in employment practice is related to the availability of sufficient cheap labor: when the price of local labor increased due to local workers’ increased access to more profitable livelihood opportunity in urban areas, the plantation owners turned to cross-border workers from Vietnam, who are introduced by informal brokers and their relatives and friends and who work for a much lower wage. Thus, the current production and harvesting of these small-scale sugarcane plantations, particularly in the regions that lie close to the border, rely heavily on seasonal migrant workers from Vietnam. It is estimated that the Vietnamese seasonal workers fill a labor shortage of around 50,000 workers during the harvest season in C city in Gunagxi (Wei 2014). However, this situation is not static but is shaped by social and political dynamics. Because of tight border controls during the pandemic, it became difficult for the plantation owners to access cheap cross-border labor from Vietnam. Instead, they had to go back to employing local villagers, particularly those who were not able to get to the urban areas during this period, with the result that labor costs on the plantations almost doubled, as shown in Table 4.

**Table 4.** Labor price of sugarcane cutters in Guangxi.

	Place of origin	Labor price
Mid-1990s	A County; T County	40–50 yuan/day
2010	Guizhou, Yunnan	1.2 yuan/bundle
2011–2019	Vietnam	1.0–1.5 yuan/bundle
Covid-19 pandemic period	Local villagers	2.2 yuan/bundle

## 4. Discussion

Based on the analysis above, there are three points that we want to highlight here in an attempt to contribute to a better understanding of plantations beyond the Chinese context, in terms of (1) scale, (2) labor relations, and (3) fundamental conditions.

### 4.1. Small-scale plantations?

The case of sugarcane plantations in Guangxi shows that plantations can be small in scale. However, this does not mean that cultivation of a cash crop on a small land plot can always be considered a plantation. As the analysis above demonstrates, particular conditions apply to the Guangxi case. Firstly, under the special zoning scheme, the land-use decisions of those operating the plantations are strongly influenced by sugar companies with the intervention of the state. Secondly, production within the zone is closely supervised by the sugar company, from the choosing of seedlings to the use of fertilizers and other inputs. Thirdly, the cutting process – particularly the timing and quantity of cutting – is fully controlled by the sugar company via the sugarcane ticket system. Finally, after cutting, the plantation owner is not free to sell the sugarcane to whoever offers the highest price, but is obliged to sell it to the company, following a set-price system. Thus, all the small-scale plantations are following the same trajectory of production, harvesting and processing, under the strict control of a single company in the zone. In other words, these plantations in Guangxi, although small when measured individually, are bound together to act as one large plantation under the zoning scheme. In this sense, the many small-scale plantations essentially form one large-scale plantation. Shaped by the particular institutional conditions of rural China, they reach the scale of operation that is attractive to a corporation, but in a form that is different from plantations in Brazil, Indonesia and elsewhere (Wolford 2004; Li and Semedi 2021)

We therefore need to rethink the question of *scale* with regard to plantations, or, more precisely, to reconsider what ‘large-scale’ really means for a plantation. Large-scale plantations are not necessarily synonymous with monocropping on a single, large tranche of land. Rather, they could comprise numerous small and fragmented areas that are organized and controlled by a single corporation for purposes of extraction. Thus, for a better understanding of the scale of a plantation, a close examination of social relations, and particularly power dynamics, is required.

### 4.2. Plantations relying on migrant labor?

This case study shows that the small-scale sugarcane plantations in Guangxi, similar to other plantations the world over, are highly dependent on migrant labor, particularly during the cutting season. However, looking into the changes in labor practices deployed in these sugarcane plantations reveals a more complicated set of dynamics.

In Guangxi, the sugarcane plantations were first dependent on family labor and labor exchange within the community, based on the principle of reciprocity. Later, with the advent of more lucrative livelihood opportunities from internal migration and the tendency towards labor commodification in rural China (Zhang 2012), wage workers were employed to fill the labor gap in the harvest season, although family labor is still largely used during the production stages. Increasing labor costs then led to local workers being replaced by migrant workers from Vietnam; as the cheapest laborers available, these Vietnamese seasonal workers became the main source of labor during the cutting season in the small-scale sugarcane plantations. Such changes are not one way, however, but can and do change further, as has been observed during the pandemic.^17^

Hence, rather than arguing that plantations rely on forced migrant labor, as has been the case historically in the plantations in California and the Pacific islands (Mitchell 1996; Cushman 2013), this paper demonstrates that plantations may be built on the cheapest source of labor that capital can access under certain institutional settings.

### 4.3. Plantations without dispossession?

This case also demonstrates that plantations do not necessarily involve direct dispossession. To put it more precisely, dispossession – namely, separating planters from land – is a sufficient, but not a necessary condition for the construction and operation of plantations. In many cases, dispossession offers a channel for corporations to acquire cheap land and labor on a large scale; in a few cases, however, it is not economic for corporations to dispossess villagers and separate them from their means of production. On the one hand, in rural Guangxi, because farmland plots are highly fragmented under the rural land system, the transaction costs of directly acquiring land to reach the scale that corporations desire can be high. On the other hand, a traditional contract farming scheme might be cheap in terms of direct land access, but the cost for corporations to maintain their control over land can be much higher considering the risks of this scheme (e.g. contract violation) (Zhang 2012; Hambloch 2022). It is thus a cheaper alternative to leave villagers on the land and incorporate them into the production process, using a zoning regime to reduce the risks of contract farming and to secure the monopoly control of corporations over the resulting plantations. Creating a ‘reserve army’ of labor is not feasible in China given the huge demand in urban areas, which has attracted vast numbers of rural laborers to become migrant workers within China. It is therefore a sound economic choice to use cross-border migrant labor from Vietnam combined with the exploitation of family labor. In this sense, plantations might be constructed and operated without dispossession.

However, this does not mean that villagers prosper. Plantations, albeit without dispossession, are extractive in nature. Although these small-scale plantation owners are not directly dispossessed and still operate the plantations, the production, harvesting and marketing of their produce are firmly controlled by sugar companies via the economic and extra-economic channels underlying the zoning regime. Moreover, under this regime, the lion’s share of profit is reaped by corporations through their direct and indirect control over cheap land and labor, while the plantation owners and migrant workers are left with few options and remain marginalized. These dynamics align with the features of extractivism discussed by Ye et al. (2020). In short, this reminds us that the fundamental feature of plantations is not dispossession, but constant extraction of value via using cheap land and labor. In this sense, it is important to focus not on dispossession, but rather on the mechanisms of investors’ extraction under specific institutional settings.

## 5. Conclusion

Sugarcane plantations in Guangxi demonstrate distinctive landscape and land–labor dynamics because of the institutional conditions in which they are set. Firstly, as a result of Guangxi’s unique rural land system, these plantations are not built on large stretches of land, but comprise small and scattered plots. Secondly, because of the high transaction costs of farmland acquisition in this context, villagers are not dispossessed but are included, and become the *de jure* owners of these plantations. But this does not mean that the villagers have *de facto* control over the plantations. Sugar companies are able to manage the production, harvesting and marketing of the plantations’ outputs, and to retain the majority of the profit from them, based on the zoning regime and with the intervention of the local state. Thirdly, combined with family labor, migrant labor from Vietnam is extensively employed to fill the labor gap in the cutting season caused by massive internal migration.

Based on these dynamics, this paper argues that although the Plantationocene has been criticized for being too broad, the concept is still of theoretical and empirical significance because it highlights the extractive nature of plantations and speaks to the power relations around control over natural resources (including land) and labor (Wolford 2021). Such a focus on the nature of power and control over plantations is able to capture the dynamics of the land–labor nexus at both the micro and macro levels. At the same time, this paper aims to empirically broaden the concept of the Plantationocene by engaging with the discussions around the scale, dispossession, and extraction. It demonstrates that the development of plantations is not always based on the monocropping on a single, large tranche of land and on the alienation of land and labor, which is distinct from the dynamics of those typical plantations in the lusotropics and Indonesia (Wolford 2021; Li and Semedi 2021). In spite of their distinctive landscape and land–labor relations, the sugarcane plantations in China discussed here are, in essence, constructed and operated under the same plantation logic as other plantations all over the world – namely, the logic of extraction, using cheap land and labor (as already included in the work of Tsing 2012; Wolford 2021; Li and Semedi 2021). That plantations could take the form of multiple small-scale plots, and could come into being without dispossession, reminds us of the need to examine the specific institutional settings in which plantations are embedded and to rethink the boundaries and contours of the plantations involved in the concept of the Plantationocene.

## Footnotes

1 In this paper, following Don Mitchell's (1996) concept, the making of landscape involves both spatiality and social integration.

2 In this paper, we use pseudonyms for the names of the townships and counties where we did our research. Meanwhile, those interviews in 2020 are conducted virtually due to the restrictions of the Covid-19 pandemic.

3 There is a decrease of sugarcane production area from 2012 to 2016. This is related to the decrease of sugarcane price which is directly link to a plummet of domestic sugar price. According the relevant news, the sugar had decreased around 44% from 8000 yuan per ton in 2011 to around 4500yuan per ton in 2015 (CCTV 2012 and He Wenting 2015). But both the sugar companies and local state actors have taken actions to retain the zoning region (e.g., offering subsidies and setting lowest sugarcane price annually). As a result, the decrease of the sugarcane area is much smaller compared with the drop of sugar price.

4 According to the Land Matrix database, the top three sugarcane plantations in terms of the size of land deals in operation worldwide are 175,000 ha in Ethiopia, 15,000 ha in Zambia and 14,733 ha in Ethiopia (https://landmatrix.org/list/deals).

5 Although main products and byproducts might change, in line with the politics of flex crops (Borras et al. 2016).

6 Although there are also relatively large-scale plantations that are run by specialized enterprises under the ‘double high’ program promoted by the Guangxi provincial government, these account for less than 10% of the total, according to authors’ interviews (field notes, 9 September 2017).

7 In this paper, the cost of controlling over land includes not only the direct cost of land acquisitions, but also the indirect cost of maintaining land control.

8 However, contract farming alone is not sufficient to be considered as a form of plantation. Because contract farming is of high risk of contract violation and thus cannot always secure investors’ control over land-use, production, and circulation of the products. So, it can be viewed as a form of plantation only when it is supplemented with schemes that secure investors’ constant extraction of value via a firm control of cheap land and labour.

9 after 2008, contracting rights were split further from the user rights with the promotion of land transfers (Ye 2015).

10 The differing capabilities is related to rural households’ distinct access to resources (mainly labour resource and social resources)

11 But this does not mean that villagers are able to completely control the land. The interventions from above, from below and the interactions of the two serve to affect the land control (for more details, see Xu 2019).

12 ‘Double high’ is a state-led program established in 2014 to promote large-scale mechanized sugarcane production with the aim of ensuring both high-quality and high-quantity outputs in Guangxi (Liu 2015).

13 Prior to 2018, the price of sugarcane in Guangxi was not determined purely by corporations but also involved state intervention. However, since 2018, the local state has stopped offering a directive price for sugarcane, giving more power to the corporations to control prices.

14 For more details, see Jiang (2014).

15 See TAKE-PROFIT.ORG

16 Although such channels have been blocked temporally due to the impacts of the pandemic.

17 Such dynamics have also been observed in sugarcane plantations in Yunnan, where seasonal migrant workers from Myanmar were largely employed (Borras et al. 2022).
